# Recent Articles—Skeletal Muscle and Other Topics in Diabetes

**DOI:** 10.1111/1753-0407.70169

**Published:** 2025-11-18

**Authors:** Zachary Bloomgarden

**Affiliations:** ^1^ Department of Medicine, Division of Endocrinology, Diabetes and Bone Disease Icahn School of Medicine at Mount Sinai New York New York USA

This commentary summarizes recently published research, giving several important findings. Deficiency of skeletal muscle appears, like excess of visceral fat, to strongly contribute to the development of diabetes. Concerns about GLP‐1RA in contributing to low muscle mass may be overstated, with the benefits of the class appearing to outweigh adverse effects; however, approaches to prevention of muscle loss with GLP‐1RA and with diabetes itself are important. Low muscle strength is a strong predictor of mortality and frailty; and resistance exercise may be a key approach in patient management. Cardiovascular risks differ between type 1 diabetes (T1D) and type 2 diabetes (T2D), with risk reduction important for both.

Obesity treatments (semaglutide, tirzepatide, and bariatric surgery) generally lower the risk of obesity‐related cancers, but bariatric surgery may increase the risk for procedure‐related cancers. Metabolic syndrome raises the risk of developing Parkinson's disease. Both hypoglycemia and higher levels of HbA1c are associated with the development of dementia.

## 1 Skeletal Muscle and Metabolic Disease

In analysis of development of T2D among participants with prediabetes in the UK biobank, the 12% with sarcopenia at baseline, based on lower handgrip strength, muscle mass and walking pace, had 19.3% versus 14.9% T2D rates over a mean 11.4 year‐follow‐up. Those with sarcopenia had, on average, slightly higher baseline BMI, but interestingly the association of sarcopenia with T2D was particularly great among those with waist: hip ratio < 0.9, suggesting sarcopenia to be an additional factor, like excess visceral fat, in causing development of T2D [1].

Botte and coworkers offer an interesting perspective on the role of low muscle strength (related both to low muscle mass and low muscle quality) in metabolic disorders [2]. Given the recognition of the importance of sarcopenia, it is instructive to summarize portions of their analysis. Low muscle strength is associated with ~2.5‐fold greater all‐cause mortality [3, 4, 5]. With aging, myosteatosis may have a particularly great impact on muscle strength, predicting fall risk and all‐cause mortality more than muscle mass in older adults [6]. BMI correlates with higher appendicular lean mass, but not with grip strength [7]. Potential sarcopenia treatment approaches include selective androgen receptor modulators (SARMS) [8], growth hormone secretagogues [9], ghrelin agonists, and fast‐twitch skeletal muscle troponin inhibitors [10], but at present none of these appear satisfactory. Recent efforts have targeted the myostatin pathway, which physiologically leads to inhibition of muscle growth [11]. Bimagrumab, an activin receptor antagonist, promotes skeletal muscle growth and fat loss, and may have benefits for bone health. While activins are also involved in reproductive hormone regulation, the selectivity of bimagrumab for ActRIIB and its pharmacokinetics appear to limit its impact on reproductive hormone pathways [12]. However, although bimagrumab is associated with an increase in lean mass it does not appear to improve functional capacity, either in strength or in physical performance [13, 14]. In a large study of non‐obese persons > 70 years of age, bimagrumab increased lean body mass and reduced fat mass, but without increasing measures of ambulation, and side effects included diarrhea, muscle spasms, and elevations in pancreatic and liver enzymes [15]. Both tirzepatide [16] and semaglutide [17] are associated with improved muscle function, and administration of liraglutide along with resistance training is associated with increased lean mass along with weight loss [18]. Thus, the incretin receptor activators, particularly when given in combination with resistance exercise, may offer an optimal approach to the combination of obesity and sarcopenia.

Pandey et al. analyzed the effects of semaglutide versus placebo on 1145 patients with heart failure with preserved ejection fraction (HFpEF), finding that the greatest improvements in quality of life with semaglutide were found in the subset of patients with the highest levels of frailty [19]. The implication is that, rather than frailty being a relative contraindication to the use of a potent GLP‐1RA because of concern that such patients would have more side effects or greater degrees of muscle loss leading to weakness, frailty appears to emerge as a particularly strong indication for the use of these medications.

## 2 Additional Topics

In 14.6 year follow‐up of 467 200 participants in the UK Biobank, of whom 177 407 had metabolic syndrome (MS) based on waist circumference, hypertension, low HDL, high triglyceride, or hyperglycemia, 3222 developed Parkinson's Disease (PD); MS was associated with a 39% increase in PD adjusted for clinical factors and a polygenic score of PD risk, with the risk proportional to the number of MS components present [20].

Patsoukaki et al. reported a 5‐year follow‐up of 38 351 adults with type 1 diabetes (T1D) and 365 675 with T2D in the Swedish national register, reporting cardiovascular disease event rates and mortality and their relationship to CV risk factors [21]. Myocardial infarction (MI) prior to age 50 was 54% more likely in T2D than in T1D, but at ages 50–59, 60–69, and 70+ years MI was 17%, 33%, and 35% less likely in T2D than in T1D, respectively. Heart failure prior to age 50 was 60% more likely in T2D than in T1D, but at age 50 or more the risks were similar for T1D and T2D. Stroke was 9% less likely in T2D than in T1D overall, and CV mortality was 19%, 25%, and 20% less likely in T2D than in T1D at ages 50–59, 60–69, and 70+ years, respectively. Further analysis revealed specific CVD associations of individual risk factors. There was 35% lower CVD risk among women. HbA1c of 7.7 was associated with 24% greater overall CVD risk than HbA1c of 6.3, with a 34% increase in risk of MI but a 14% increase in CVD mortality. Higher LDL cholesterol was associated with a particularly great increase in MI but, surprisingly, with lower heart failure and CVD mortality risks. A major conclusion of the analysis was that CVD risk reduction should be considered as important for T1D as for T2D, particularly at older age groups, leading the authors to opine that studies of cardiorenal benefits of SGLT2 inhibitors, GLP‐1 receptor agonists, and mineralocorticoid antagonists should be undertaken in populations with T1D as has been done for T2D.

An interesting study looked at rates of obesity‐associated cancers (OAC) (of the breast, colorectum, gallbladder, liver, multiple myeloma, esophageal, ovarian, pancreatic, renal, gastric cardia, thyroid, and uterine) in propensity score‐adjusted controls treated with a dipeptidyl peptidase 4 inhibitor (DPP4i) compared to those treated with semaglutide (64 178 matched pairs, 1.5 year follow‐up), with tirzepatide (19 682 matched pairs, 0.8 year follow‐up), and with gastric bypass surgery (9642 matched pairs, 4.9‐year follow‐up) in the TriNetX electronic health records network [22]. Total OAC likelihood was 12% lower with semaglutide, 16% (but not significantly) lower with tirzepatide, and 15% lower with bariatric surgery. For specific sites, with semaglutide colorectal cancer decreased 20%, liver cancer 25%, and pancreatic cancer 24%; with tirzepatide ovarian cancer decreased 69%; and with bariatric surgery liver cancer decreased 44%, uterine cancer decreased 41%, but esophageal and gastric cardia cancer increased 4.8‐ and 10.5‐fold, respectively. Given the relatively short follow‐up with the GLP‐1RA and the small numbers of individual cancers, the results may be subject to bias, with the authors suggesting that physicians might have been less likely to start these agents in patients with early symptoms subsequently associated with malignancy, but the findings for total OAC do support the recognition that obesity is associated with malignancy, and suggest that measures to reduce obesity may be of benefit in reducing malignancy. Another finding of concern was the increased esophageal and gastric cardia cancers with bariatric surgery, compatible with a worrisome complication of these procedures.

A meta‐analysis of 40 studies representing 7 076 724 people with diabetes showed that those with a history of hypoglycemia had a 1.5‐fold increase in likelihood of dementia, with comparable findings for Alzheimer's disease and vascular dementia, and with one study showing an association of dementia with greater numbers of hypoglycemic episodes [23]. Higher levels of HbA1c were associated with greater likelihood of dementia. The studies examining HbA1c variability and those with information about diabetes duration showed these factors to be associated with greater likelihood of dementia as well.

## Conflicts of Interest

The author declares no conflicts of interest.
